# Intraprocedural Artificial Intelligence for Colorectal Cancer Detection and Characterisation in Endoscopy and Laparoscopy

**DOI:** 10.1177/1553350621997761

**Published:** 2021-02-26

**Authors:** Niall P Hardy, Pól Mac Aonghusa, Peter M Neary, Ronan A Cahill

**Affiliations:** 1 UCD Centre for Precision Surgery, School of Medicine, 58041 University College Dublin, Dublin, Ireland; 2 Healthcare Research, 535630 IBM Research, Dublin, Ireland.; 3 Department of Surgery, University College Cork, 8797 University Hospital Waterford, Waterford, Ireland; 4 Department of Surgery, 8881 Mater Misericordiae University Hospital, Dublin, Ireland

**Keywords:** artificial intelligence, fluorescence-guided surgery, colorectal disease, machine learning, decision support systems, biophysics-inspired algorithms

## Abstract

In this article, we provide an evidence-based primer of current tools and evolving concepts in the area of intraprocedural artificial intelligence (AI) methods in colonoscopy and laparoscopy as a ‘procedure companion’, with specific focus on colorectal cancer recognition and characterisation. These interventions are both likely beneficiaries from an impending rapid phase in technical and technological evolution. The domains where AI is most likely to impact are explored as well as the methodological pitfalls pertaining to AI methods. Such issues include the need for large volumes of data to train AI systems, questions surrounding false positive rates, explainability and interpretability as well as recent concerns surrounding instabilities in current deep learning (DL) models. The area of biophysics-inspired models, a potential remedy to some of these pitfalls, is explored as it could allow our understanding of the fundamental physiological differences between tissue types to be exploited in real time with the help of computer-assisted interpretation. Right now, such models can include data collected from dynamic fluorescence imaging in surgery to characterise lesions by their biology reducing the number of cases needed to build a reliable and interpretable classification system. Furthermore, instead of focussing on image-by-image analysis, such systems could analyse in a continuous fashion, more akin to how we view procedures in real life and make decisions in a manner more comparable to human decision-making. Synergistical approaches can ensure AI methods usefully embed within practice thus safeguarding against collapse of this exciting field of investigation as another ‘boom and bust’ cycle of AI endeavour.

“Technology, through automation and artificial intelligence is one of the most disruptive sources of our age. It changes the way we work and the skills we need”

Alain Dehaze, CEO the Adecco Group

## Introduction

Minimal access interventions provide screen-based display of internal appearances for human practitioner interpretation to guide intellectual and mechanical progress through a complex procedure. Since its inception in the 1960s, such videoscopy has revolutionised our ability to diagnose, monitor and manage an abundance of gastrointestinal conditions at sites otherwise inaccessible without traditional operation.^1^ Both colonoscopy and laparoscopy are deployed for the diagnosis and treatment of disease of the colon and rectum and require high levels of interventionalist cognition and dexterity for the correct, confident labelling of abnormalities encountered in the absence of hard landmarks. Both interventions provide the opportunity over diagnostic radiology to directly sample and even cure lesions encountered, ideally at the index procedure. To do so requires real-time human realisation of abnormality and a qualitative decision based on training and experience to act as part of the perception/action neural loop in the interventionalist’s brain. Surgical decision-making is particularly rooted in the opinion and judgement of the human expert with less objective visual aids to prompt or justify actions than those available to the endoscopist.^2^

Before now, advances have predominantly either been in the field of hardware optimisation (e.g. big screen display with resolution up to 4K, technological adjuncts such as 3D and augmented surface characterisation including narrow band spectral reading and real-time microscopic examination of colorectal mucosa in situ^3,4^) or through procedural organisation (e.g. technical standardisation and specialisation with centralisation of certain patient cohorts such as screening populations).^5^ Challenges remain though as both colonoscopy and laparoscopy are fundamentally operator-dependent procedures. This is compounded by patients with colorectal disease who ultimately require specialist input presenting to a variety of healthcare settings both electively and emergently, and so their initial care is via a spectrum of practitioners with differing levels of expertise and technological capability. Artificial intelligence (AI) methods offer the opportunity to augment human interpretation intraprocedurally and to provide statistical measures of the relevance of lesions as they are encountered in real time, including all contributory data around the case (e.g. pathology and radiology).^6^ Logically, these can progress to decision support systems for critical steps whether removal, in situ ablation or accelerated, streamlined further investigation to ensure the right patient gets exactly the right level of care improving interventional accuracy and minimising wasteful over-investigation. Modern-day computer vision (CV) techniques offer several potential benefits to a surgeon (see Table 1) although some limitations with regard to newer computational interpretations have recently become apparent especially with respect to deep learning (DL) that questions their suitability in the field of medicine where ‘explainability’, ‘interpretability’ and accountability are of paramount importance. These issues are reviewed here extrapolating learnings from recent endoscopic advances into the laparoscopic paradigm and alternative or indeed complementary methods, such as biophysics-inspired computer modelling, discussed to frame the near-term evolution of this exciting field for surgeons.

**Table 1.** Offerings of computer vision capability to laparoscopic surgery.

Capability	Benefit
Ability to constantly monitor and detect subtle on-screen patterns that may be missed by, or imperceptible to, the human eye	Addresses issues of consistency and cognitive burden in surgical practice by adding a tireless, ever-present computerised assistant
Development and training of machine learning systems to the highest possible standard and their worldwide availability with quantitative decision support based on statistical models capturing many examples of previous practice as standard practice leading to better standards of care	Augmentation of the qualitative decision-making of a single operator
Convergence of radiology and pathology data streams integrated alongside surgical intraoperative visual findings	Allows more dynamic analysis of on-screen findings to prompt appropriate actions to be taken by the operator in real time

### AI and Machine Learning

It is generally accepted that modern AI as a concept first arose at a meeting in Dartmouth College, Hanover, in 1956 which simply challenged: ‘Every aspect of learning or any other feature of intelligence can be so precisely described that a machine can be made to simulate it’.^7^ Computer scientists, the world over set about designing such ‘intelligent’ computer systems although it was decades before increased computing power and data storage capabilities, along with the emergence of big data, enabled their implementation to become mainstream. AI approaches initially focussed on logical, knowledge-based approaches – the so-called von Neumann architectures – where knowledge is programmed as decision rules. Many decision rules are aggregated together to attempt to cover every anticipated scenario, often resulting in large, complex collections of rules. Although well suited for situations where possible scenarios are limited, such as controlling the movements of a manufacturing robot, such approaches do not scale well to other tasks curtailing the optimism of these early and the so-called ‘Golden Years’ of AI.^8^

The 1990s saw a resurgence in AI research with advancements so significant that this period is now referred to as the ‘first AI revolution’ with standout examples of computers surpassing even brilliant human minds in specific tasks (e.g. IBM’s Deep Blue defeat of chess Grand Master Garry Kasparov).^9^ Rapidly, computers were applied to areas such as speech and facial recognition, internet search engines and image classification.^10^ Machine learning (ML), whereby an AI system can be programmed to learn from example data (and not decision rules) to perform classification and prediction tasks, marked a break from traditional knowledge-based approaches. DL then evolved in the mid-2010s. Increased computing capabilities and algorithmic advances, for example, back propagation, meant learning algorithms consisting of many networked layers of interconnected processing units known as neurons could be designed as neural networks ((NNs), first conceived in the 1940s), in principle, similar to human NN whereby inputted data are passed through several complex hidden steps to extract patterns from data at scale arriving at an output. Inclusion of a mathematical operation called convolution, a specialised kind of linear operation, in place of general matrix multiplication in at least one layer of a NN opens DL application to visual imagery in the form of a convolutional neural network (CNN).

The use of DL is growing rapidly across many areas of medicine. Specific DL architectures such as CNNs have successfully performed image recognition tasks such as detection of diabetic retinopathy and cutaneous melanoma and the classification of breast lesions.^11‐13^ Deep learning however requires a large corpus of examples (commonly tens of thousands of examples) for training and testing. The ground breaking DeepMind project in ophthalmology required one million retinal scans to achieve its results.^14^ This requirement limits the application of DL to all, but the most common procedures where archives of large numbers exist from routine application. Techniques to reduce the dependence of learning algorithms on large volumes of data are an active area of research in the ML community.^15^

Other methods of AI exist, for example, specific mathematical modelling techniques such as biophysics-inspired modelling (BIM) which builds on understanding of the dynamics of biological and physical processes to describe them in terms of a simplified number of parameters. For instance, perfusion of blood through tissue is well described in the literature using a range of so-called compartment models which reduce the complex underlying advection and diffusion processes into a small number of physical parameters.^16^ Simplifying the description of complex biophysical processes of interest in this way can help develop accurate predictive techniques, with confidence intervals, without dependence on vast image banks. Recent studies of angiogenesis demonstrate how a fundamental understanding of cell biology, gained through traditional experimental models, can be combined with mathematical/computational modelling to explore the spatial and temporal aspects of vessel replication in new ways.^16^

### Endoscopy vs Laparoscopy

AI methods so can likely play a role in assisting interventionalist orientation via continuous re-evaluation of the procedural field in real time to draw the operator’s attention to the important clinical regions and help cancel out unhelpful surrounding noise. While the endoscopist and surgeon are generally performing a similar visually driven act, the spectrum of signals and potential actions precipitated is much broader for the surgeon.

#### Endoscopy for colorectal cancer

Studies have consistently proven that adenomatous polyps represent potentially cancerous precursor lesions and that their removal is positively associated with reduced colorectal cancer rates.^1^ Arguably therefore the role of colonoscopy in the detection, characterisation and resection of these lesions is more valuable than its role in diagnosing cancer and underpins its worldwide adoption. 1% increases in adenoma detection rates (ADRs) at colonoscopy have shown 3-6% reductions in interval cancer rates.^1,17,18^ However ADRs vary greatly between institutions for numerous reasons including endoscopist experience, withdrawal time and the number of individuals observing the monitor during procedures.^19,20^ While imaging quality advances such as narrow band imaging (NBI) and chromoendoscopy, as well as regular auditing of key performance indicators have improved standards worldwide, these measures are inconsistently implemented between centres.^4^ Along with detection, lesion/tissue characterisation is an essential attribute of effective colonoscopy. While it remains important to detect and adequately resect all sessile serrated or adenomatous polyps, there exist many diminutive non-neoplastic polyps, especially in the rectosigmoid region, that are of no clinical relevance.^21,22^ The removal of these lesions places a large burden on histopathology services as well as putting patients at risk of undue harm from unnecessary polypectomy. Aside from leaving innocuous lesions, confidence in the correct categorisation of lesions that do need address accelerates the patient towards definitive care.

#### Laparoscopy for colorectal cancer

In the last 20 years, much elective operation for colorectal cancer is now commenced and completed laparoscopically. The advantages to this approach are well proven in terms of short-term convalescence, and it is now the standard access of choice.^23,24^ Robotic-assisted systems provide electromechanical platforms that enable greater precision at instrument tips. Neither method has yet augmented intraoperative decision-making other than providing improved visualisation. Therefore, safe and effective surgery depends crucially on the surgeon’s ability to recognise structures in the field of vision and to plot the operative sequence from initiation through to conclusion. Similar to colonoscopy and different to other surgical fields such as orthopaedics, there is a lack of rigid measures of orientation and therefore, it is difficult to provide means of anatomical direction and mile-stoning other than via surgeon expertise with continuous checking to reassure. Inter-individual variation, prior surgery, disease and obesity can challenge anatomical recognition including fascial plane, neurological structure and adjacent organ identification.

In particular, classification of lesions unrecognised by preoperative imaging and seen for the first time at surgery currently relies on the subjective assessment of the operator. The peritoneum along with the mesocolonic, mesenteric and liver surfaces is often poorly characterised on computerised tomographic imaging, yet any lesions present here affect the staging of the patients and impact theranostically.^25^ Frozen section provides a degree of intraprocedural assistance to the operator in certain circumstances; however, it is not always available and may not be definitive with small, fragile tissue samples. Similar so to colonoscopy, an ability to detect and characterise lesions and recognise major normal anatomy automatically is crucial, especially in high stakes decision-making during major operation where it needs to be rapid and highly accurate. However, the field of view is more complex.

### AI Methods in Endoscopy

As a commonly performed procedure readily suitable to image capture, AI methods have developed in endoscopy and are now commercially available (for example: GI Genius, Medtronic. MN, USA) concentrated predominantly in the areas of lesion detection and characterisation and, more recently, are providing assistance in quality measures such as bowel preparation and withdrawal times.^26,27^ Early exploits involved rudimentary AI methods performing retrospective analysis of initially static, followed by dynamic, images.^28‐31^ These methods provided acceptable sensitivities and specificities in post hoc static image testing that then fell sharply when attempts were made at real-time analysis.^32-34^ Furthermore, the desire to ensure detection of all lesions encountered came at the price of unacceptably high false positive rates.^35^ The emergence of big data and CNNs saw the resurgence of AI methods in endoscopy as well as near real-time decision-making and reduced false positive rates.^33,36‐38^

As is the natural progression in emerging technologies, small pilot studies have paved the way for larger randomised trials. *Wang et al.*^39^ recently published on 1058 patients randomised to either standard or computer aided colonoscopy with a significant increase in adenoma detection per patient being seen (.31 vs .53, *P* <.001). Even more impressively, *Su et al.*^26^ reported data from a randomised study of 623 patients whereby 315 patients were allocated to conventional, unassisted colonoscopy and 308 patients assigned to their automatic quality control system (AQCS). This system incorporated 5 Deep CNN models to automatically time scope withdrawal (triggered by the systems recognition of the caecum), detect polyps (adenomatous and non-adenomatous) as well as assessing bowel preparation dynamically after system training on data from 4000 patients with white light images labelled by two gastrointestinal experts. This is the first time that AI methods monitored such important quality measures with the user being prompted to suction debris or slow down withdrawal rate when appropriate. Fundamentally, this approach represents a shift away from unidimensional structure (polyp) recognition to a computer system acting as a type of ‘procedure companion’ that accompanies the operator and provides active guidance throughout the journey. The authors reported statistically significant increased rates of total polyp (.383 vs .254) as well as adenoma (.289 vs .165) detection rates in their AQCS study arm as well as significantly increased scope withdrawal times (7.03 vs 5.68 minutes). False prompt rates of .21 prompts per colonoscopy were reported in this study. As is a common feature throughout AI methods for polyp detection false positives remain a challenging aspect for AI methods to overcome. *Hassan et al.* in their report quote ‘negligible false positive rates’ of .9% false positive frames in their first validation study. They also acknowledge however that real-life data will consist of roughly 50,000 frames per colonoscopy suggesting that this false positive frame rate will likely remain notable in clinical practice.^40^

#### AI methods in surgery

AI in surgery is not at all as advanced as in colonoscopy. This is likely due to the increased complexity and heterogeneity of structures and elements in any field of view combined with the general lack of similar annotated video banks for exploitation. Where it does exist, it is currently deployed for operative video segmentation and provision of crude measures of operative fluency such as measuring the time the camera is in the interior, instrument profiling and partitioning of an operation into its major steps.^41^ Increasing the contrast in the field of view, especially as it may relate to either critical structure normal anatomy (for preservation) or disease identification (for removal), as has been seen in the field of fluorescence-guided surgery (FGS) is ideal terrain for CV application.^42,43^ This combines extended spectral imaging with exogenous fluorophore administration (predominantly indocyanine green – (ICG)^44‐46^ to disclose information regarding the nature of the tissues being seen by the visualisable presence of contrast dye in the region of interest such as perfusion characterisation or biliary anatomy identification (ICG circulates in the blood stream before being excreted unchanged in the bile).^47^ Ureters have been similarly visualised using methylene blue (an agent excreted selectively by the kidneys) and a shorter wavelength illumination (c700 nm vs 780 for ICG) although progress to routine clinical use is being held up due to licencing issues related to the agent.^48^ There are many additional agents in development and some even in phase 2 clinical trials that aim to further advance the field in terms of structure specificity and ease of identification.^49^

With such added contrast, AI has a promising role to play in providing means of objectively quantifying dye presence, especially if needed to do so kinetically, that is, determine the rate of filling or emptying of contrast from the region of interest. This would potentially add much information and ease use of many agents which right now are predicated upon administration long before surgery in order to aid human identification (i.e. aiming to target the window of maximum presence in the area of concern with minimal background elsewhere). By trying to create a static, coloured field, the techniques of FGS are prone to false positives and error especially if the timing is different to what had originally been planned (operative lists are variable in terms of exact procedure times). Usefulness is limited however if agents must be pre-planned and administered days before surgery rather than being used simply at the point of necessary enquiry. Furthermore, because much information is known regarding dye and illumination energy behaviour in tissue, this can be factored into AI algorithms by exploiting BIM to characterise the underlying video signal in terms of well-known physical parameters related to diffusion and advection. A particular attraction of the biophysics-inspired approach is that it alleviates the requirements for millions of images in the training bank as is typical in pure ML or DL approaches. This method then does not rely on the analysis and comparison of surface appearance alone but instead seeks to delve into the very biology of the tissue in question. In the realm of fluorescent-guided surgery, AI sensitivity may allow less specificity in agent development as the fluorescent agent will no longer have to do all the work in lesion identification (nor indeed will the human in its recognition).

The ability to characterise lesions based on their biology dramatically reduces the number of cases needed to build a reliable system and instead of focussing on image by image analysis could analyse in a continuous fashion, more akin to how we view the procedures in real life. Furthermore, identification of tissue based on its true biology, rather than on attempted identification by patterns such as shape or colour as used in the DL methods seen in endoscopy, will address the still present issue of false positives. We have already proved this concept in primary colorectal cancer using computer-aided interpretation of minute differences in dynamic perfusion patterns between the distorted architecture of neoplastic tissue and that of the surrounding ‘normal’ tissue. Our experimental study of this BIM on a corpus of 20 colorectal cancer endoscopic videos correctly identified 19/20 (95%) lesions with 100% cancer sensitivity (91.7% specificity).^50,51^

### Limitations and Future Directions

Artificial intelligence methods promise to significantly boost human decision-making in terms of image recognition and are already making inroads into soft tissue endoscopy moving beyond rigid object (like that seen with self-driving cars) and defined margin (that used for melanoma photographs and mammograms) analysis.^10,12,13^ The advent of DL/NN has improved research results for colorectal lesion characterisation and detection; however, these systems fundamentally rely on large banks of reference images from which they ‘learn’. To increase system performance, more training images need to be acquired. Such ‘polyp maps’ are created through analysis of, in some cases, millions of images to detect new similar appearing lesions among images previously unseen to the system with studies using ‘in-house’ databases of reference images. This limits comparison of results across studies undermining generalizability. Also, the need for large volumes of images increasingly presents moral challenges as well as logistical ones. These concerns relate to data ownership and patient privacy, particularly in the cases of ‘for profit’ systems. The ‘black box’ nature of these complex systems may also understandably raise similar concerns. In many cases, the exact workings of these systems remain incompletely understood yet are earmarked for widespread use in the delivery of modern healthcare in the near future. In the early stages of clinical integration, the concept of ‘explainability’ of results generated from such systems is a crucial component of adoption by healthcare providers and insurers. This differs from ‘interpretability’ which relates to the extent to which a cause and effect can be observed within a system. Furthermore, *Antun et al.* recently highlighted the inherent instability of DL because of the processes it uses to reconstruct and store images.^52^ Imperfect image reconstruction methods that are accentuated with increasing volumes of images collected may lead to computational errors.

In contrast, BIM has the advantage over other AI techniques when it comes to ‘interpretability’. Their decision processes build their foundation upon known biological phenomena which results in a more predictable pattern of decision-making and more closely replicates the human minds decision processes. For decisions in surgery and even endoscopy, especially where dynamic modelling of tissues may be helpful, the use of fluorescence and computer-aided decision augmentation may help to smooth the differences in operator ability from centre to centre using computer classifiers that are easily interpreted to assist the operator in their endeavours to provide the highest level of care to patients. Furthermore, computer processes could provide alternative presentation of data through mathematical modelling such as 2D and even 3D maps of the tumour. Such AI systems that analyse in real time based on tissue biology also obviate the need for the collection of large masses of patient data for training purposes and, if successful, would have implications across all disciplines of healthcare. Furthermore, such in vivo contrast enhancement can help train AI systems on the corresponding white light imagery.

## Conclusion

While the era of AI and computer augmented decision-making in medicine is still very much in its infancy, it likely portends a significant revolution in improving the standard of healthcare delivered worldwide, and it is developing rapidly. The increasingly widespread availability of technology allows more research groups than ever access to this rapidly evolving area and thus further promotes improvements. Daunting barriers to use on a large scale remain, however, most notably the high rate of false positives, the large volumes of comparative pictures that are currently required to ‘educate’ these computer systems and the difficulties associated with explaining and accounting for unseen, and poorly understood, processes happening within some AI models. Correct clinical integration also needs consideration likely in the first phases as collaborative systems rather than challenging the role of the doctor as decision-maker with the likely advent of a new type of practitioner, the interventionalist (be it gastroenterologist or surgical technologist). Importantly too, the history of computer science teaches us that, whatever the AI methodology, always unforeseen pitfalls arise leading to ‘boom and bust’ cycles further encouraging complementary methods of advance to safeguard against collapse of this exciting field of investigation.
